# Impact of the crosstalk between the PTEN and PAFR as well as PAFR and EGFR pathways in cancer

**DOI:** 10.37349/eds.2025.100883

**Published:** 2025-01-14

**Authors:** Anita Thyagarajan, Zaid Sirhan, Ravi P. Sahu

**Affiliations:** Department of Pharmacology and Toxicology, Boonshoft School of Medicine at Wright State University, Dayton, OH 45435, USA

**Keywords:** Phosphatase and tensin homolog, platelet-activating factor-receptor, epidermal growth factor receptor, cell signaling pathways, cancer therapy

## Abstract

The integration between the tumor-suppressive and oncogenic signaling pathways controls various cellular activities of cancer cells, including cell growth and apoptosis. While the activation of oncogenes fuels cancer progression and escape mechanisms, tumor suppressors regulate and counterbalance the negative effects of oncogenic signaling. Notably, phosphatase and tensin homolog (PTEN) constitute one of the important family members of tumor suppressor genes, which play critical roles in regulating the activities of tumor cells. Thus, an impaired, mutated, or loss of PTEN is associated with low survival or high tumor recurrence rates in cancer patients. Importantly, high tumor expression of a G-protein coupled platelet-activating factor-receptor (PAFR) is associated with increased tumor progression as well as decreased overall survival and poor prognosis in malignancies such as non-small cell lung cancer (NSCLC). Along similar lines, overactivation or mutations in epidermal growth factor receptor (EGFR) signaling are detected in various human malignancies and associated with poor prognosis. The goal of the current minireview was to highlight the significance of the mechanistic insights between the PTEN and PAFR as well as the PAFR and EGFR pathways in impacting cancer growth and/or efficacy of therapeutic agents in experimental model systems.

## Introduction

As integration between the tumor suppressive and oncogenic signaling pathways regulate cancer cell activities, including cell growth and apoptosis, understanding the mechanistic insights will provide approaches to overcome drug resistance and improve the efficacy of therapeutic agents. Among tumor suppressor family members, the phosphatase and tensin homolog (PTEN) plays an important role in mediating lipid phosphatase activity that antagonizes phosphatidylinositol 3-kinase (PI3K) resulting in the inhibition of the downstream mammalian target of the rapamycin (mTOR)/AKT signaling pathway [1–3]. The PTEN functions have been extensively studied in various cell culture systems, preclinical models, and clinical settings of malignancies, including lung cancer [4–6], and reviewed in reference [7]. Notably, reduced expression or loss of tumoral PTEN is associated with the overall low survival of cancer patients. Thus, approaches to overcome this effect would aid in the ongoing challenges of cancer treatment, including non-small cell lung cancer (NSCLC) [4, 6, 7]. As PTEN remains an undruggable target, strategies to modulate its activity via targeting counteracting pathways such as platelet-activating factor-receptor (PAFR) signaling are being explored as potential approaches to overcome therapeutic resistance.

The PAFR signaling plays critical roles in various physiological and pathophysiological conditions, including cancer [8–11]. Notably, clinical studies demonstrated that high tumoral-PAFR expression correlates with increasing tumor stages/invasiveness, poor prognosis, and decreased overall survival in lung and esophageal squamous cell carcinoma patients [12, 13]. Of significance, increased levels of PAF agonists or PAFR activity have been detected in perfusates and tumor samples collected post-chemotherapy and post-radiation therapy compared to pre-chemotherapy and pre-radiation therapy in melanoma and non-melanoma patients [9, 14]. Along similar lines, da Silva-Jr et al. [8] reported high levels of PAFR expression in tumor samples collected from cervical invasive carcinoma patients treated with radiotherapy compared to untreated patients. This indicated that understanding the insights into the PAFR pathway and its crosstalk with potential signaling mechanisms such as epidermal growth factor receptor (EGFR) could provide potential target(s) to be explored against malignancies, including NSCLC.

Importantly, receptor tyrosine kinases (RTK) such as EGFR signaling play critical roles in modulating the cellular activities of tumor cells, including cell proliferation [15, 16]. Its overactivation or mutations also account for the acquired tumor resistance and/or reduced efficacy of tyrosine kinase inhibitors (TKIs) in preclinical experimental models, and cancer patients, reviewed in references [16, 17]. The generations of EGFR-TKIs are based on their characteristics and the order they were developed [18]. For example, the first-generation EGFR-TKIs are reversible EGFR inhibitors such as erlotinib and gefitinib. The second generation includes irreversible erythroblastic leukemia viral oncogene homolog (ErbB) family blockers, including EGFR such as afatinib and dacomitinib, which were designed to overcome resistance to first-generation EGFR-TKIs. The third-generation EGFR-TKIs include irreversible EGFR mutant selective and wild-type (WT) EGFR sparing such as osimertinib for patients with T790M acquired resistant mutation [19]. However, patients can develop resistance to these EGFR-TKIs via mechanisms mediated by both EGFR-dependent and EGFR-independent pathways [20, 21]. While there was ample evidence about the roles and mechanisms of the PTEN, PAFR, and EGFR pathways, there was no published review article that highlights the impact of the crosstalk between the PTEN and PAFR as well as the PAFR and EGFR pathways in cancer models, which was the goal of this minireview. As PTEN inactivation is associated with resistance to EGFR-TKIs, we anticipated that PAFR could be involved as a central mechanism in mediating inactivated PTEN-induced EGFR overactivation, and thus, could be exploited as a potential target to overcome EGFR-TKIs resistance.

## PAF and PAFR pathway

The generation of reactive oxygen species (ROS) is one of the common mechanisms of pro-oxidative stressors, including radiation therapy and chemotherapeutic agents [8, 9]. Studies, including ours, provided compelling evidence that such ROS-generating agents, produce oxidized phospholipid mediators, PAF, and PAF-like agonists, which bind to and activate a G-protein coupled PAFR, expressed on a variety of cell types, including tumor cells [8, 9, 22–24]. Besides, PAF agonists are also synthesized via highly regulated remodeling and de novo pathways involving cytosolic phospholipase A_2_ (cPLA_2_), acetyltransferase, and acetylhydrolase enzymes [22–24]. The roles and mechanisms of the PAF-PAFR signaling in mediating acute pro-inflammatory and delayed chronic responses, including systemic immunosuppression and cancer growth have been studied and reviewed in references [10, 11]. The immune cell types implicated in PAF-PAFR mediated systemic immunosuppressive effects include CD4+CD25+FoxP3+ regulatory T cells (Tregs), CD11b+Gr-1+ myeloid-derived suppressor cells (MDSCs), and CD163+, CD206+, Arg1+ tumor-associated macrophages (TAMs) [8, 9, 24, 25]. Notably, these immunosuppressive cell types also play critical roles in augmenting tumor growth or impeding the efficacy of therapeutic agents in experimental cancer models, including NSCLC [8, 9, 12, 25, 26]. Importantly, pharmacological inhibitors of cyclooxygenase type 2 (COX-2) or depleting antibodies against Tregs attenuate PAFR-mediated systemic immunosuppression, and tumor growth, as well as enhance the efficacy of chemotherapeutic agents and radiation therapy in experimental cancer models [9, 14]. These experimental findings indicated that therapeutic agents can produce PAF agonists, which mediate pro-tumoral responses, and impede the efficacy of cancer therapies in a PAFR-dependent manner.

Given that PAF-metabolizing, PAF-acetylhydrolase (PAF-AH) can readily metabolize PAF agonists, a question as to how PAF agonists mediate delayed systemic immunosuppression and pro-tumoral effects remained unanswered. To that end, our recent studies determined the significance of a subpopulation of extracellular vesicles referred to as microvesicles, large extracellular vesicles, or microvesicle particles (MVPs). Our studies demonstrated that gemcitabine treatment for PANC-1 pancreatic cancer cells generates MVPs in a PAFR-dependent manner and that these MVPs contain PAF agonists [27]. Along similar lines, we have shown that targeted therapies, gefitinib and erlotinib treatment to A549 and H1299 NSCLC cell lines produce MVP release in a PAFR-dependent manner, in a process blocked by PAFR antagonist or PAFR silencing [28]. As the biosynthesis of MVP requires the activation and translocation of an acid sphingomyelinase (aSMase), our studies demonstrated that an aSMase-specific inhibitor, imipramine blocks targeted therapy-induced MVP release in NSCLC cells [28]. These findings highlighted the relevance of PAFR signaling in therapeutic agents-induced MVP release, which acts as a novel mechanism by which metabolically labile PAF agonists are not only protected but circulated to exert local as well as delayed systemic effects. Importantly, as PTEN crosstalks with several oncogenic signaling cascades to regulate the growth/fate of tumor cells, including NSCLC, herein, we discussed the key findings, highlighting the crosstalk of the PTEN and PAFR pathways, and PAFR and EGFR pathways and their significance in cancer and cancer therapies.

### Crosstalk between the PTEN and PAFR pathways

The evidence that PTEN interacts with the PAFR pathway came from the earlier report demonstrating that treatment with a specific PAFR antagonist, WEB2086 to breast carcinoma MCF-7 and MDA-MB-231 cell lines causes G0/G1 cell cycle arrest [29]. This effect was accompanied by functional changes, including decreased invasive behavior of these cell lines upon treatment with the WEB2086 compound. These WEB2086-induced effects were found to be mediated via an increased expression of PTEN [29]. Nevertheless, as both MCF-7 and MDA-MB-231 cell lines express endogenous PAFR [30] and WEB2086 upregulates PTEN, these findings supported the possible crosstalk between the PTEN and PAFR pathways and indicated that PAFR suppresses PTEN expression via a negative feedback loop.

**Figure 1.**
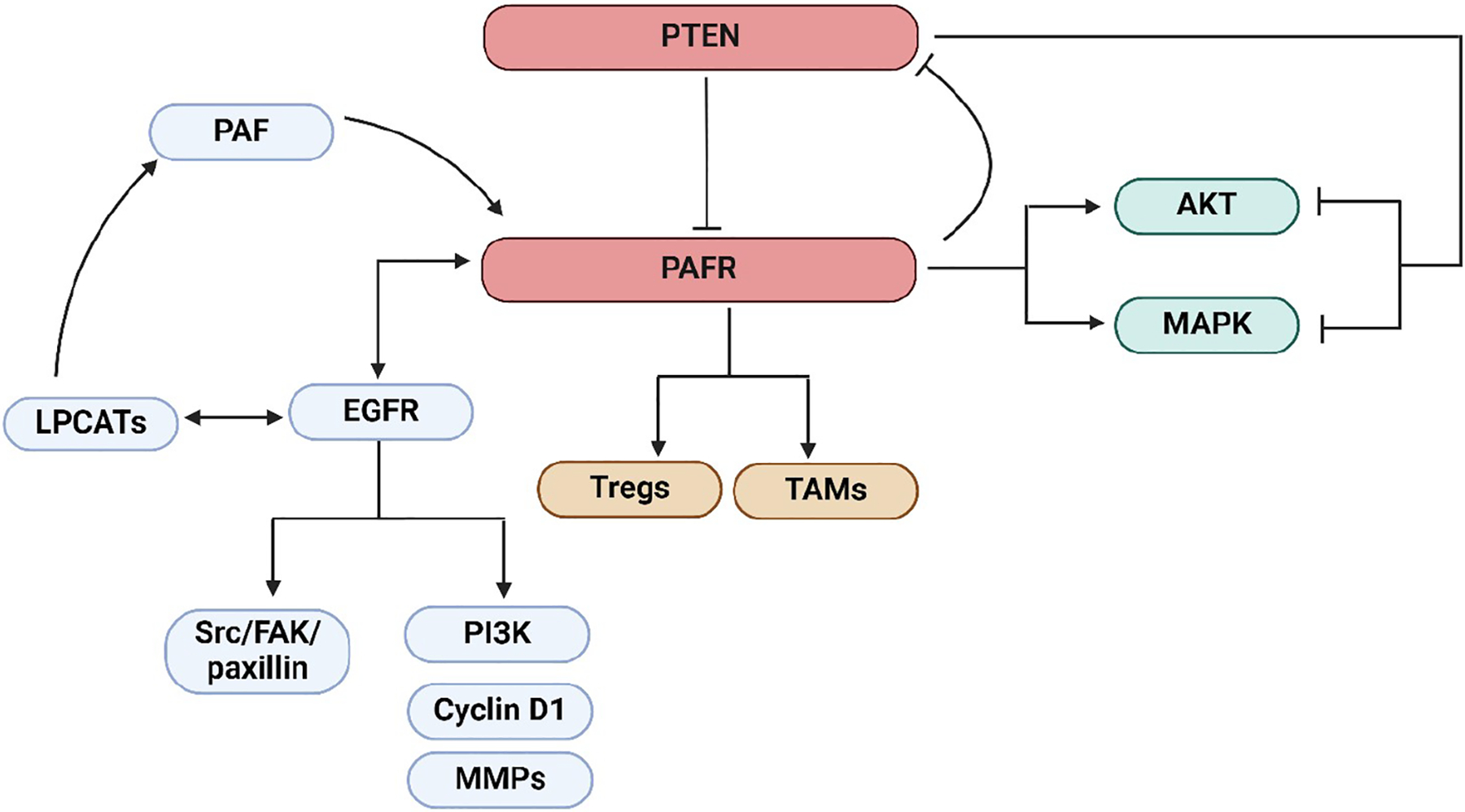
The schematic representation of the crosstalk between the PTEN and PAFR as well as PAFR and EGFR pathways resulting in the activation of LPCATs enzymes generating PAF, or the downstream signaling cascades, Src/FAK/paxillin or PI3K, cyclin D1, and MMPs. PTEN inhibits the PAFR signaling. The activation of the PAFR signaling by PAF agonists results in the upregulation of immunosuppressive cell types, Tregs, and TAMs. The PAFR suppresses PTEN expression via a negative feedback mechanism. Also, PAF activates the downstream signaling cascades, MAPK and AKT, in the process blocked by PTEN. Altogether, these mechanisms can lead to the augmentation of tumor growth or inhibition of cancer therapy efficacy. PTEN: phosphatase and tensin homolog; PAFR: platelet-activating factor-receptor; EGFR: epidermal growth factor receptor; LPCATs: lysophosphatidylcholine acyltransferases; PI3K: phosphatidylinositol 3-kinase; Src: proto-oncogene tyrosine-protein kinase; Tregs: regulatory T cells; TAMs: tumor-associated macrophages; MAPK: mitogen-activated protein kinase; MMPs: matrix metalloproteinases; FAK: focal adhesion kinase. Created in BioRender. Sirhan, Z. (2025) https://BioRender.com/q65n763

Later, Kim et al. [31] provided supporting evidence of the crosstalk between the PTEN and PAFR pathways via studies demonstrating that PAF-induced increased pulmonary metastasis of B16F10 melanoma tumor was blocked by adenovirus harboring cDNA construct of PTEN (Ad-PTEN). Importantly, treatment of PAF induced the phosphorylation of the downstream mitogen-activated protein kinase (MAPK) family members [i.e., extracellular-regulated protein kinase (ERK), P38, and c-Jun N-terminal kinase (JNK)], and AKT pathways, which was blocked by Ad-PTEN. This indicated the potential mechanism by which PTEN not only directly inhibited PAFR’s function but also targeted its downstream signaling cascades [31]. Notably, as cigarette smoking generates PAF agonists, a recent clinical study conducted in cohorts of smoking and non-smoking bladder cancer (BC) patients demonstrated that smoking BC patients had a higher frequency of mutations in 9 cancer-related genes, including *PTEN* compared to non-smoking BC patients [32]. Along similar lines, other studies, unrelated to cancer have also confirmed the interaction between PTEN and PAF [33]. The schematic representation of signaling cascades that mediated the crosstalk between the PTEN and PAFR as well as the PAFR and EGFR (detailed below) pathways was shown in Figure 1. The summary of the crosstalk between the PTEN and PAFR pathways was given in Table 1.

### Crosstalk between the PAFR and EGFR pathways

As PAFR activation induces gefitinib and erlotinib (which inhibit the tyrosine kinase activity of the EGFR) induced MVP release [28], it is important to understand the insights into the interplay between the PAFR and EGFR axis in impacting cancer growth and/or efficacy of therapeutic agents, to devise novel approaches to improve therapy effectiveness. The first evidence indicating the crosstalk between the PAFR and EGFR pathways came from the earlier report demonstrating that PAF treatment to ovarian cancer cell lines caused an increased phosphorylation of EGFR and its downstream signaling axis Src (proto-oncogene tyrosine-protein kinase)/FAK (focal adhesion kinase)/paxillin as well as the activation of PI3K and cyclin D1, which are involved in cell proliferation, and matrix metalloproteinases (MMPs) (MMP2 and MMP9), which are involved in cell invasion [34]. Therefore, these effects resulted in increased proliferation and invasion of OVCA 429 and OVCA 432 ovarian cancer cell lines in a process blocked by PAFR antagonist ginkgolide B and PAFR-specific antibody, as well as erlotinib; Src TKIs, PP2 and A25; and PI3K inhibitor, LY294002 [34]. This crosstalk was later confirmed by Yu et al. [35], demonstrating that PAF-PAFR-dependent increased phosphorylation of EGFR in SKOV3 ovarian cancer cell line was mediated via phospholipase C-β (PLCβ) and intracellular Ca^2+^ signaling, and dependent on Src tyrosine kinase and metalloproteinases.

Later, the same group examined the synergistic effects of targeting the PAFR and EGFR signaling on the antitumor efficacy of ovarian cancer [36]. The authors found that a combination of PAFR antagonist (WEB2086) and EGFR inhibitor (AG1478) resulted in significantly increased inhibition of proliferation and invasion of CAOV3 and SKOV3 cell lines, as well as decreased growth of CAOV3 tumor xenografts in athymic nude mice compared to these drugs alone [36]. Along similar lines, the same group demonstrated that treatment with EGF augments PAF production in CAOV3 and SKOV3 cell lines via increased phosphorylation of ERK, and was blocked by the inhibition of EGFR, PAFR, and cPLA_2_ enzyme involved in PAF synthesis [37]. Importantly, EGF-induced increased PAF production was found to be mediated via transactivation of the PAFR, which was blocked by EGFR inhibition [37].

Notably, Yao et al. [38] demonstrated that ultraviolet B (UVB)-induced increased generation of ROS and PAFR agonists from epidermal KB cells, as well as systemic immunosuppression, was blocked by EGFR inhibitor PD168393 treatment. Exploring The Cancer Genome Atlas (TCGA) database of cervical cancer specimens, Souza et al. [39] reported a strong positive correlation between the PAFR and EGFR, and EGFR and lysophosphatidylcholine acyltransferases (LPCATs), which were involved in PAF biosynthesis. Moreover, EGFR activation increases PAFR and LPCAT2 expression, and a significantly greater effect was noted in highly aggressive ovarian cancer CASKI cells compared to less aggressive C33A cells. Notably, PAF treatment caused EGFR transactivation in the CASKI cell line leading to increased ERK activation and COX-2 induction. Importantly, inhibition of the PAFR by WEB2086 and EGFR by cetuximab resulted in decreased cell viability and inhibition of the colony-forming ability of CASKI cells [39]. Of importance, LPCAT2 activation has also been shown to enhance PAF production in lipopolysaccharide-stimulated macrophages [40]. The summary of the crosstalk between the PAFR and EGFR pathways was given in Table 1.

**Figure 2.**
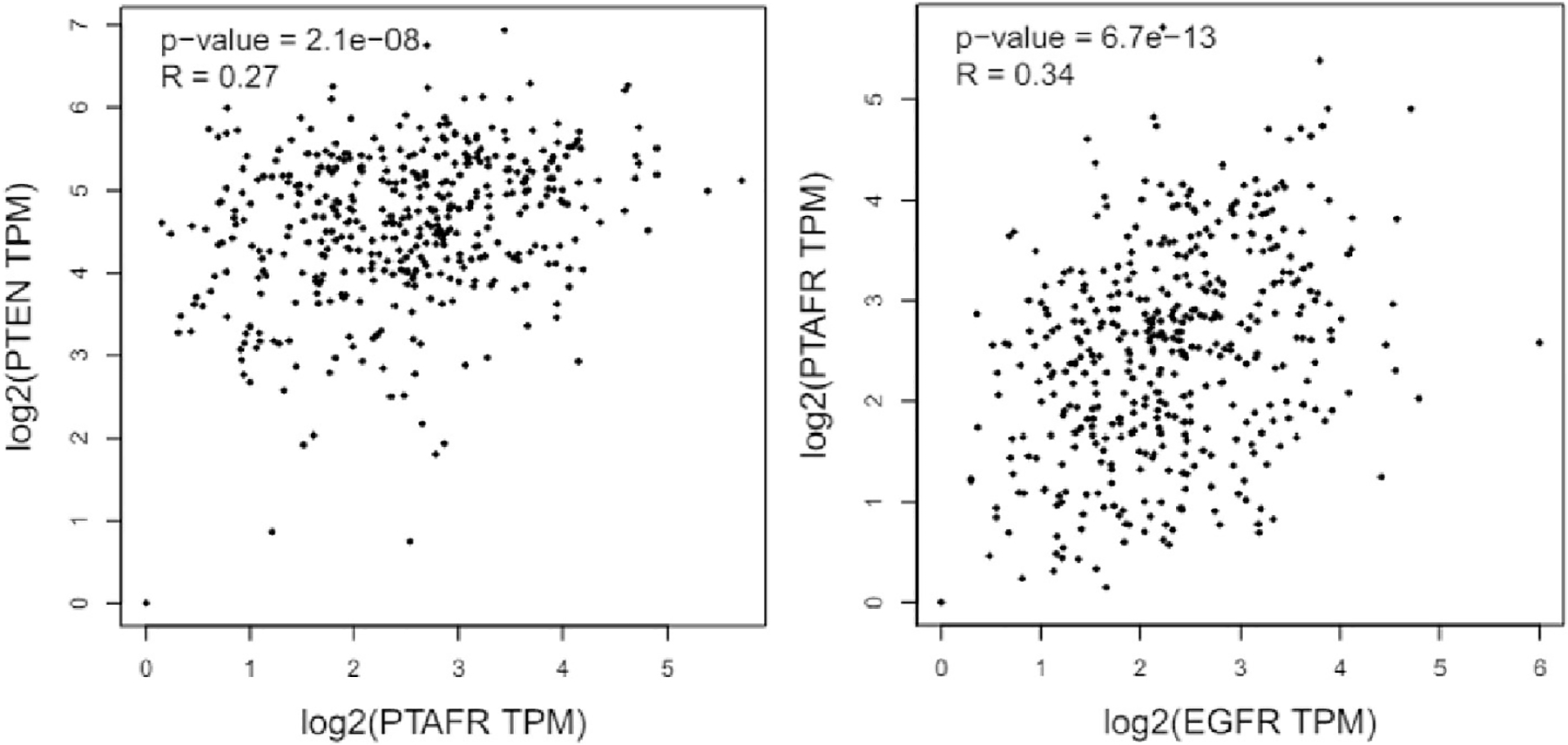
Bioinformatics analysis using the TCGA GEPIA2 database indicated the correlation between the PTEN and PAFR, and PAFR and EGFR in ovarian serous cystadenocarcinomas. PTEN: phosphatase and tensin homolog; PTAFR: phosphatase activating factor-receptor; EGFR: epidermal growth factor receptor; TPM: transcripts per million; TCGA: The Cancer Genome Atlas; GEPIA2: Gene Expression Profiling Interactive Analysis 2

To further support the crosstalk between the pathways, we used the GEPIA2 (Gene Expression Profiling Interactive Analysis 2) database [41] and conducted a correlation analysis. We selected ovarian serous cystadenocarcinoma (OV) from the “TCGA tumor database” as most of the experimental studies used ovarian cancer models. While the Spearman correlation coefficient (i.e., *R* value) indexes of around 0.3 do not strongly indicate correlations, this does suggest a modest interaction between the PTEN and PAFR [i.e., *PTAFR* (platelet-activating factor-receptor)] and the PAFR and EGFR pathways (Figure 2).

## Conclusions

PTEN targets the PAFR signaling, which interacts with EGFR and its downstream cascades, and PAFR/EGFR blockade has been shown to inhibit the growth of tumor cells. While the interaction between the PTEN, PAFR, and EGFR pathways remains elusive, the consummated findings provide compelling evidence that the PTEN-PAFR and PAFR-EGFR axis (and their downstream cascades) represent novel targets for malignancies, including NSCLC. Given that malignant cells develop resistance to currently used EGFR-TKIs, and PAFR can mediate inactivated PTEN-induced EGFR overactivation, targeting PAFR could be exploited as a potential strategy to overcome EGFR-TKIs resistance in cancer patients having altered/mutated EGFR. Overall, these findings suggested that pharmacological approaches to target these pathways could also be explored to mitigate therapy-induced adverse events and enhance therapy effectiveness.

## Figures and Tables

**Table 1.** Summary of studies demonstrating crosstalks between the PTEN and PAFR as well as PAFR and EGFR pathways

Cancer model	Cell line(s)	Treatment(s)	Key findings	Reference
Breast cancer	MCF-7, MDA-MB-231	PAFR antagonist	PAFR antagonist-mediated decreased invasive behavior and G0/G1 cell cycle arrest was mediated via increased PTEN expression	[29]
Melanoma	B16F10	Ad-PTEN	Ad-PTEN blocked PAF-induced pulmonary melanoma metastasis	[31]
Ovarian cancer	OVCA 429, OVCA 432	PAFR antagonist	PAFR antagonist blocked PAF-induced activation of EGFR and downstream signaling cascades resulting in decreased cell proliferation	[34]
Ovarian cancer	SKOV3	-	PAF-PAFR signaling induced increased EGFR activation	[35]
Ovarian cancer	CAOV3, SKOV3	PAFR antagonist and EGFR inhibitor	Increased inhibition of cell proliferation and invasion and decreased tumor growth	[36]
Ovarian cancer	CAOV3, SKOV3	PAFR antagonist and EGFR inhibitor	Inhibition of the PAFR and EGFR blocked EGF-induced PAF production	[37]
Epidermal cells	KB	EGFR inhibitor	Inhibition of increased production of PAFR agonists and systemic immunosuppression	[38]
Ovarian cancer	CASKI, C33A	PAFR antagonist and EGFR inhibitor	Decreased cell viability and proliferation	[39]

PTEN: phosphatase and tensin homolog; PAFR: platelet-activating factor-receptor; EGFR: epidermal growth factor receptor; Ad-PTEN: adenovirus harboring cDNA construct of PTEN; -: no data

## Data Availability

The datasets analyzed in Figure 2 can be found in the TCGA GEPIA2 http://gepia.cancer-pku.cn/.

## Abbreviations

Ad-PTEN: adenovirus harboring cDNA construct of phosphatase and tensin homolog
aSMase: acid sphingomyelinase
BC: bladder cancer
cPLA_2_: cytosolic phospholipase A_2_
COX-2: cyclooxygenase type 2
EGFR: epidermal growth factor receptor
ERK: extracellular-regulated protein kinase
LPCATs: lysophosphatidylcholine acyltransferases
MMPs: matrix metalloproteinases
MVPs: microvesicle particles
NSCLC: non-small cell lung cancer
PAFR: platelet-activating factor-receptor
PI3K: phosphatidylinositol 3-kinase
PTEN: phosphatase and tensin homolog
ROS: reactive oxygen species
Src: proto-oncogene tyrosine-protein kinase
TCGA: The Cancer Genome Atlas
TKIs: tyrosine kinase inhibitors
Tregs: regulatory T cells
